# Supplementary material for: The role of telemedicine towards improved sustainability in healthcare and societal productivity in Turkey
Source: PLoS One. 2024 Dec 5;19(12):e0314986. doi: 10.1371/journal.pone.0314986 (PMC11620697; doi:10.1371/journal.pone.0314986)
Supplement: Attachment — Submitted filename: Response_to_Reviewers.pdf [file pone.0314986.s001.pdf]

July 12th, 2024

Prof. Boyen Huang, DDS, MHA, PhD
Academic Editor
PLOS ONE

RE: **PONE-D-24-12793** "The role of telemedicine towards improved sustainability in health care and societal productivity in Turkey"

Dear Professor Huang:

We would like to thank you, the editorial board and reviewers for their valuable comments on our manuscript titled *"THE ROLE OF TELEMEDICINE TOWARDS IMPROVED SUSTAINABILITY IN HEALTHCARE AND SOCIETAL PRODUCTIVITY IN TURKEY"*. Hereby, we are submitting a revised version of our work and providing point-by-point responses to the reviewers' comments. We hope that our work will satisfy the acceptance criteria.

Sincerely,
Dilsun Kaynar

Prof. Huang:

Please submit your revised manuscript by Jul 26, 2024, 11:59PM.

Please include the following items when submitting your revised manuscript:

- A rebuttal letter that responds to each point raised by the academic editor and reviewer(s). You should upload this letter as a separate file labeled 'Response to Reviewers'.
- A marked-up copy of your manuscript that highlights changes made to the original version. You should upload this as a separate file labeled 'Revised Manuscript with Track Changes'.
- An unmarked version of your revised paper without tracked changes. You should upload this as a separate file labeled 'Manuscript'.

Response: *All documents have been uploaded.*

Critique: We note that you have indicated that there are restrictions to data sharing for this study. For studies involving human research participant data or other sensitive data, we encourage authors to share de-identified or anonymized data. However, when data cannot be publicly shared for ethical reasons, we allow authors to make their data sets available upon request.

Before we proceed with your manuscript, please address the following prompts:

a) If there are ethical or legal restrictions on sharing a de-identified data set, please explain them in detail (e.g., data contain potentially identifying or sensitive patient information, data are owned by a third-party organization, etc.) and who has imposed them (e.g., a Research Ethics Committee or Institutional Review Board, etc.). Please also provide contact information for a data access committee, ethics committee, or other institutional body to which data requests may be sent.

b) If there are no restrictions, please upload the minimal anonymized data set necessary to replicate your study findings to a stable, public repository and provide us with the relevant URLs, DOIs, or accession numbers. Please see http://www.bmj.com/content/340/bmj.c181.long for guidelines on how to de-identify and prepare clinical data for publication. For a list of recommended repositories, please see https://journals.plos.org/plosone/s/recommended-repositories. You also have the option of uploading the data as Supporting Information files, but we would recommend depositing data directly to a data repository if possible.

Please update your Data Availability statement in the submission form accordingly.
Response: *We changed the data sharing to anonymized data sets available upon request.*

Critique: Please include your full ethics statement in the 'Methods' section of your manuscript file. In your statement, please include the full name of the IRB or ethics committee who approved or waived your study, as well as whether or not you obtained informed written or verbal consent. If consent was waived for your study, please include this information in your statement as well.
Response: *We updated the Materials and Methods section to indicate that consent was waived for this study and information on the ethics committee is also given in that section.*

Critique: We note that Figure 4 in your submission contain map images which may be copyrighted. All PLOS content is published under the Creative Commons Attribution License (CC BY 4.0), which means that the manuscript, images, and Supporting Information files will be freely available online, and any third party is permitted to access, download, copy, distribute, and use these materials in any way, even commercially, with proper attribution. For these reasons, we cannot publish previously copyrighted maps or satellite images created using proprietary data, such as Google software (Google Maps, Street View, and Earth). For more information, see our copyright guidelines: http://journals.plos.org/plosone/s/licenses-and-copyright.

We require you to either (1) present written permission from the copyright holder to publish these figures specifically under the CC BY 4.0 license, or (2) remove the figures from your submission:

Response: *We removed Figures 1-3 per reviewer suggestions and renamed Figure 4 as Figure 1. Current Figure 1 is intended to convey the same information as the old Figure 4 and it has been obtained from* https://earthexplorer.usgs.gov, *which is a public domain.*

Group comments

We thank the reviewers for their constructive feedback, which led us to rewrite the introduction and make several key changes to make the thesis of our paper clearer. We state below how our revision addresses reviewer comments in an itemized fashion.

Reviewer #1:

Critique: The topic is important and timely. The manuscript is mostly well written, although it could be shortened quite a bit and better structured. The discussion should be more focused and to the point- especially the start. Figures 1-5 are unnecessary.

Response: *Thank you for the input. We significantly edited the manuscript, by making its focus clearer and removing Figures 1-3.*

Critique: ..., the analyses, interpretations and conclusions are probably too positive with regard to telemedicine. The assumption that all visits after the first visit could be performed with telemedicine is highly problematic. Further, health-risks and quality are not discussed and the estimation of work-hours is not realistic- e.g. not all patients are employed. Thus, the manuscript does not comprehensively present a realistic scenario regarding cost/benefits, risks and actual implementation. This should receive more attention.

Response: *We rewrote several sections of the manuscript making a clearer distinction between our conclusions for the specific context of our study (specific CVD diagnoses) based on our dataset and what we think about the potential benefits of telemedicine in general. We also expanded the Discussion and Limitations sections to include potential problems with telemedicine in more detail.*

We acknowledge the critique about work-hours assumption applying to all patients. Therefore, we incorporated a new reference to a manuscript by Stratmann and modified our claim to say ".... -unless the visit leads to gain in health and subsequent productivity [62–67]." (Line 274). *Moreover, we added discussion of this issue in the Limitations section.*

Critique: The major weakness concern the assertion of all visits post first visit can be done with telemedicine. This should at least be ameliorated, as this is directly relevant for the estimates.

Response: *We respectfully believe that the top 5% diagnoses such as venous insufficiency used to build our model can safely be managed through telemedicine after the first in-person visit.*

Reviewer #2:

Critique: The paper is on an interesting topic, looking into the role of telemedicine and whether the use of this technology could help in saving time and reduce the carbon footprint. The authors could expand more on the background session.
Response: *We restructured and rewrote several sections, including more references as appropriate.*

Critique: Methodology part is clear and well presented, as well as the discussion. It is good that you looking into the carbon footprint and the economic gains of using telemedicine, but it would be useful to look also in the quality of these consultations, the intangible qualities of telemedicine. I don't know for example, if you are aware of the paper by Chonglin Sun et al. on Telemedicine Practice for Social and Environmental Sustainability and other works on telemedicine and sustainable social development.
Response: *As we did not have the actual telemedicine consultations, we did not have the actual telemedicine data, however we made further discussion points based on related work, including the work by Dr. Sun et al.*

Reviewer #3:
Critique: In the abstract the cost savings were for clinic visits yet it reads that cost savings may occur from hospital visits. How do you know that hospital visits may have occurred?
Response: *In this manuscript, we used the words clinic and hospital interchangeably, meaning physical locations to which patients travel for receiving medical care.*

Critique: The first two sentences of the abstract are illogical--COVID is over so how can telemedicine alleviate the current burden?
Response: *We removed the COVID statement from the abstract.*

Critique: The abstract has too many errors to note. The authors toggle between the terms telehealth and telemedicine--they are different and consistency is needed. What does recent mean? Two minutes ago, two decades ago? Asynchronous is not always pre-recorded information. Wage loss in confusing. You seem to be assuming everyone is working yet some are retired, on disability, etc. Why are the ages for males <60 and females <58? Is there a lower limit?
Response: *We carefully revised the manuscript, making consistent references to telemedicine, time frames, synchronous vs. asynchronous.*

As a clarification to the reviewer, Turkey has a retirement age of 60 for males and 58 for females and that was the reason for our earlier assumptions. This was stated in the original manuscript as well as the revised one in the section "Economic impact of traveling to healthcare facilities" starting on Line 136.

Reviewer #4:
Thank you for the opportunity to read this paper the contribution of telemedicine on sustainability of health care. This paper aims to examine: i) the hypothetical impact of delivering telehealth care to patients in a busy tertiary cardiovascular clinic in Istanbul, Turkey; ii) potential environmental and societal ramifications of telemedicine. To address this issue, authors used demographics, health care costs, wages, productivity, and patient-specific data. The main goal is to develop a hypothetical telemedicine framework for the Turkish health care landscape using information related to the distance traveled and travel time to receive care using real-life location of the clinics and patients addresses.
Data from August 3, 2015 to January 25, 2023 resulting in 45,602 unique encounters with 448 unique diagnoses recorded for the patient encounters. Authors reported that 656,258 km would have been saved if all patients were to take the first visit in person followed by telemedicine visits. Telemedicine could save approximately 30% carbon footprint and prevented approximately $ 878,000 wage loss. They concluded that the application of telemedicine could ease the burden on patients, environment, increase access, and prevent the wage losses caused by unnecessary hospital visits.

Critique: The introduction should serve as a comprehensive literature review, setting the stage for your study. Unfortunately, this section is currently a major weakness in your manuscript. To strengthen it, you could consider the following points:
1. Begin by presenting an overview of cardiovascular diseases (CVDs), including their prevalence, impact, and burden on public health globally, and then narrow down to the context of Turkey. Use up-to-date statistics and references to highlight the significance of the problem.
2. Emphasize the need for innovative interventions to address the burden of CVDs. Introduce digital technologies, particularly telemedicine, as a potential solution.
3. Provide a clear definition of telemedicine and discuss its potential benefits and challenges, including barriers to adoption and existing knowledge gaps.
4. Present the relevance and potential value of a hypothetical telemedicine framework in mitigating the burden of CVDs.
Response: *We sincerely appreciate your constructive criticism. We restructured the manuscript and rewrote the introduction and revised the subsequent sections with your guidance.*

Critique: Materials and Methods: There are some ambiguities and a lack of structure in this section. For instance, I will encourage the authors to follow **standard headings and subheadings** such as: *study design, population and settings; measurement outcomes, dependent and independent variables, types of measurements, data analysis*; Could you explain the term “declaration."
Provide a detailed and systematic description of your methods to ensure reproducibility and clarity.
Response: *We restructured and edited the materials and methods sections.*

Critique: Results: The results section should be presented clearly and logically. Currently, it is challenging to understand the study's objectives and the significance of the findings. I will suggest to authors to re-organize their results in a logical manner, corresponding to your research questions or hypotheses, highlight the key findings and their relevance to the study's objectives.

Response: *We restructured the results section to follow the logical flow of data and reflect the order in the materials and methods section.*

Critique: Limitations: The limitations section needs to be more precise. Specifically, please avoid overgeneralizations, such as comparing a single department with an entire healthcare system. Authors should discuss specific limitations related to your study design, data collection, analysis, and interpretation, as well as their potential impact on your conclusion. By addressing these points, you can significantly improve the quality and impact of your manuscript. I encourage you to thoroughly revise each section to ensure clarity, coherence, and rigor.
Response: *We made major changes in our manuscript along these lines. We pay attention to making a clearer distinction between conclusions for the specific context of our study, based on our data, and what we think about the potential benefits of telemedicine in general.*
