# Supplementary material for: The role of telemedicine towards improved sustainability in healthcare and societal productivity in Turkey
Source: PLoS One. 2024 Dec 5;19(12):e0314986. doi: 10.1371/journal.pone.0314986 (PMC11620697; doi:10.1371/journal.pone.0314986)
Supplement: Attachment — Submitted filename: Response_to_Reviewers_PONE-D-24-12793R2_10302024.docx [file pone.0314986.s002.docx]

October 30^th^, 2024

**Prof. Boyen Huang, DDS, MHA, PhD**
Academic Editor
**PLOS ONE**

**RE:**	**PONE-D-24-12793R1** “The role of telemedicine towards improved sustainability in health care and societal productivity in Turkey”

Dear Professor Huang:

We would like to thank you and the editorial board and reviewers for their valuable comments and time for our revised manuscript entitled *“THE ROLE OF TELEMEDICINE TOWARDS IMPROVED SUSTAINABILITY IN HEALTHCARE AND SOCIETAL PRODUCTIVITY IN TURKEY"*. We here are submitting a new revised version of our work and are providing point-by-point responses to the reviewers’ comments in this ‘Response to Reviewers’ letter. We hope that our work will satisfy the review process.

Sincerely,

Zeliha Dilsun Kaynar, PhD

**Prof. Huang:**

Thank you for submitting your manuscript to PLOS ONE. After careful consideration, we feel that it has merit but does not fully meet PLOS ONE’s publication criteria as it currently stands. Therefore, we invite you to submit a revised version of the manuscript that addresses the points raised during the review process.

**Please be advised to address the reviewers' comments thoroughly, particularly on (1) justification of the participants' employment status, (2) recognition of the global history of telemedicine development and evolution, (3) the length of the discussion section, and (4) appropriate references.**

**Response:** Thank you, Prof. Huang, for these high-level critiques. In the rest of the document, we address each of them in detail. To summarize:

1- The employment status critique is very much appreciated for a better modeling of the wage loss. We used OECD data to refine our model.
2- We modified our writing such that we recognize the global history of telemedicine.
3- We shortened and focused the discussion.
4- We added appropriate references.

**Comments to the Author**

We thank the reviewers for their constructive feedback, which led us to rewrite the introduction and make several key changes to make the thesis of our paper clearer. We state below how our revision addresses reviewer comments in an itemized fashion.

**Reviewer #1:**

**Critique:** In the R1 version of the manuscript " The role of telemedicine towards improved sustainability in healthcare and societal productivity in Turkey", the authors have submitted a revised version and responded to reviewer comments.

While the manuscript is improved, the authors have not remedied my main objection about the assumption that all patients below pension-age are employed. Does everybody below 58 years of age in Turkey have full-time jobs? I guess not and this must be described and modelled.

**Response:** We thank the reviewer for this very important critique. To estimate the number of people who were employed before the pension age, we used the latest employment rate data from the *Organisation for Economic Co-operation and Development* (OECD), available using the links below. This data shows the average employment to be 57.4% between 2015 through 2022 for the 15-64 age range regardless of the gender.

<https://www.oecd.org/en/publications/oecd-economic-surveys-turkiye-2023_864ab2ba-en.html>

[https://data-explorer.oecd.org/vis?lc=en&df[ds]=DisseminateArchiveDMZ&df[id]=DF_DP_LIVE&df[ag]=OECD&df[vs]=&av=true&pd=2015%2C2023&dq=TUR%2BOECD..15_64..A&to[TIME_PERIOD]=false&vw=tb](https://data-explorer.oecd.org/vis?lc=en&df%5bds%5d=DisseminateArchiveDMZ&df%5bid%5d=DF_DP_LIVE&df%5bag%5d=OECD&df%5bvs%5d=&av=true&pd=2015%2C2023&dq=TUR%2BOECD..15_64..A&to%5bTIME_PERIOD%5d=false&vw=tb)

**Reviewer #3:**

**Critique:** The authors state, "Telemedicine is a recent development within healthcare." What do you mean by recent? Telemedicine has been around for decades. Figure 1 is difficult to read. May want to telehealth as a key word.

**Response:** We thank the reviewer. We use a more careful language recognizing the fact that telemedicine has been practiced for decades while still making the point that its adoption has been accelerated by COVID-19 (page 18).

We added “Telehealth” added as a key word (page 3).

**Reviewer #4:**

**Critiques:** Authors addressed many comments, and the paper has been improved. However, some points still require clarification. My main comments are on the objective/methods and discussion sections. I have minor comments on the limitation and conclusion sections.

**Critique:**

**Objective:** Our study focuses on patients with CVDs and the potential savings afforded by telemedicine in travel distance and time, and lost wages as delayed access to acute as well as chronic care is associated with increased mortality.

• The following sentences should be moved to the method section
*We also calculated 71 the hypothetically associated savings in travel distance and time for hospital visits enabled by 72 synchronous video conferences. The busy cardiology and cardio-vascular surgery clinics in 73 Istanbul used in our study can also be viewed as a model for studying the potential positive patient 74 outcomes, environmental, economic, and societal ramifications of deploying telemedicine. 75 Moreover, the location of these clinics can demonstrate the benefits of telemedicine in settings 76 where specialized care is concentrated in certain clinics in a populous city only accessible via often 77 traffic-laden travel routes. 78 We obtained datasets for demographics, healthcare costs, wages, productivity, and data of 79 patients of the above-mentioned clinics to develop a hypothetical telemedicine framework. 80 Specifically, we obtained the distance and time expended to receive care at the clinics by using the 81 addresses of the clinics and patients seeking care. The calculated distances and travel times allowed 82 us to calculate the carbon footprint and wage losses associated with traveling to seek in-person 83 care in lieu of telemedicine appointments. 84 In the case of Turkey, telemedicine acceptance was fast during the COVID-19 pandemic, yet 85 the capacity for expansion, financing, policies, governance, and partnership are critical factors for 86 any new system to be successful [22,23]. The hypothetical benefits of adopting telemedicine 87 presented in this work could inform and motivate future policies in this space.*

**Response:** The sentences have been relocated.

**Critique:**

• Could they add references here
- ”The carbon footprint of traveling by bus is 105 grams per kilometer and the carbon footprint of 165 traveling by car is 192 grams per kilometer.”
- Ref 30 is unclear, please Check for your bibliography
- As the work by Sun et al. (Year) (ref) suggests, telemedicine has the potential to alleviate some of the problems associated with the overburdened healthcare systems worldwide [38]
• I was not able to see these figures 7,8,9 in legends

**Response:** Corrected and references added

The Ref 30 in R1 was about the impact of aging society on the health care expenditure. “*The effect of population aging on health expenditure growth: a critical review. Eur J Ageing. 2013;10: 353–361. doi:10.1007/s10433-013-0280-x*”

The following phrase was deleted: “As the work by Sun et al. (Year) (ref) suggests, telemedicine has the potential to alleviate some of the problems associated with the overburdened healthcare systems worldwide [38]”

We removed the Figures 7-9 and the associated legends.

**Critique:**

Discussion
Discussion is very long and the authors are not focusing on their results. They could start their discussion at line 335. I invite them to emphasize on the results and discuss them with the literature.
*Our study focuses on patients with CVDs and the potential savings afforded by telemedicine in travel distance and time, and lost wages as delayed access to acute as well as chronic care is associated with increased mortality.
This research aims about the savings afforded to telemedicine in travel distance and time, and lost wages….*

"The authors reported the non-inferiority of telemedicine and its impact on quality-related outcomes. I would suggest they focus more closely on the specific goal of the study and avoid overgeneralization.

The term 'quality of care' is broad, and the effects of telemedicine on overall quality remain unclear. They could narrow their discussion to a few specific components of quality of care rather than addressing it as a whole”
“Our findings are supported by the recent literature that has demonstrated the non-inferiority of outcomes in telemedicine treatment compared with face-to-face treatment for patients with peripheral arterial and venous diseases [45–47]. There are published and ongoing clinical trials for personalized telemedicine around cardiovascular health [48]. A recent work compared the use of telemedicine to in-person doctor visits among patients with congestive heart failure (CHF). Among 850 patients with CHF, a hybrid telemedicine care model was not inferior to in-person visits for mortality [49]. In a trial among 1,119 patients with CHF, telemedicine-based management emergency service for high-risk HF patients proved to be safe and reduced unplanned hospitalizations [50]. Another trial showed decreased emergency medicine department admissions among patients with CHF if they were followed up by a tele-pharmacist [51]. Based on the clinical trials, there is an important and effective potential role for telemedicine in healthcare [52–54]”

Here, you could split in two sentences:
Delayed access to acute as well as chronic care is associated with increased mortality (._) and prior Some authors work showed the negative impact of long wait periods on CVD outcomes [39–41]

Limitations:
I recommended to keep 1 and 4. I am not sure th numbers 2 and 3 are relevant to your study.

Conclusion:
Your conclusion goes beyond the scope of your study. For instance, you could rewrite like this:
“This way, telemedicine could become a valuable tool for sustainable cities of the future.”

Suggestion
In the limitation of your study, further research is necessary to explore …… in order to effectively promote telemedicine as a valuable tool for developing sustainable cities.

**Response:** As can be observed in the revised manuscript, we followed the reviewer’s recommendations closely. We think that this led to a revised manuscript that is better organized, more focused and avoids over-generalizations.

7. PLOS authors have the option to publish the peer review history of their article ([what does this mean?](https://urldefense.com/v3/__https:/c05y1x9s.r.us-east-2.awstrack.me/L0/https:*2F*2Fjournals.plos.org*2Fplosone*2Fs*2Feditorial-and-peer-review-process*23loc-peer-review-history/1/010f01924c0359c1-fa75d9a3-ddff-41c1-abc1-6cbf351f632b-000000/LnfxO1x6yMwoNNyK-S2VppiIfjM=179__;JSUlJSUl!!NHLzug!KIz36wJKaBEvPCLoXD7iBPMcNgNcB5FWHqshgKeH8CB62p3QLJKkiz3nuZbZKFwrziKQJW5U2NKiBgQzYQjjaQ$)). If published, this will include your full peer review and any attached files.


If you choose “no”, your identity will remain anonymous but your review may still be made public.


**Do you want your identity to be public for this peer review?** For information about this choice, including consent withdrawal, please see our [Privacy Policy](https://urldefense.com/v3/__https:/c05y1x9s.r.us-east-2.awstrack.me/L0/https:*2F*2Fwww.plos.org*2Fprivacy-policy/1/010f01924c0359c1-fa75d9a3-ddff-41c1-abc1-6cbf351f632b-000000/bQQOu_HV7TcIgroi5fK1_rlmwh8=179__;JSUl!!NHLzug!KIz36wJKaBEvPCLoXD7iBPMcNgNcB5FWHqshgKeH8CB62p3QLJKkiz3nuZbZKFwrziKQJW5U2NKiBgReXhaQqw$).

Reviewer #1: No

Reviewer #3: **Yes:** Lois Ritter

Reviewer #4: No
